# Solid state fermentation of rapeseed cake with *Aspergillus niger* for degrading glucosinolates and upgrading nutritional value

**DOI:** 10.1186/s40104-015-0015-2

**Published:** 2015-04-08

**Authors:** Changyou Shi, Jun He, Jie Yu, Bing Yu, Zhiqing Huang, Xiangbing Mao, Ping Zheng, Daiwen Chen

**Affiliations:** Animal Nutrition Institute, Sichuan Agricultural University, Xinkang Road 46#, Ya’an, Sichuan Province 625014 People’s Republic of China

**Keywords:** *Aspergillus niger*, Glucosinolates, Nutritional value, Rapeseed cake, Solid state fermentation

## Abstract

**Background:**

Rapeseed cake is a good source of protein for animal feed but its utilization is limited due to the presence of anti-nutritional substances, such as glucosinolates (Gls), phytic acid, tannins etc. In the present study, a solid state fermentation (SSF) using *Aspergillus niger* was carried out with the purpose of degrading glucosinolates and improving the nutritional quality of rapeseed cake (RSC). The effects of medium composition and incubation conditions on the Gls content in fermented rapeseed cake (FRSC) were investigated, and chemical composition and amino acid *in vitro* digestibility of RSC substrate fermented under optimal conditions were determined.

**Results:**

After 72 h of incubation at 34°C, a 76.89% decrease in Gls of RSC was obtained in solid medium containing 70% RSC, 30% wheat bran at optimal moisture content 60% (w/w). Compared to unfermented RSC, trichloroacetic acid soluble protein (TCA-SP), crude protein and ether extract contents of the FRSC were increased (*P* < 0.05) 103.71, 23.02 and 23.54%, respectively. As expected, the contents of NDF and phytic acid declined (*P* < 0.05) by 9.12 and 44.60%, respectively. Total amino acids (TAA) and essential amino acids (EAA) contents as well as AA *in vitro* digestibility of FRSC were improved significantly (*P* < 0.05). Moreover, the enzyme activity of endoglucanase, xylanase, acid protease and phytase were increased (*P* < 0.05) during SSF.

**Conclusions:**

Our results indicate that the solid state fermentation offers an effective approach to improving the quality of proteins sources such as rapeseed cake.

## Background

Rapeseed cake (RSC) or meal is the by-product of oil extraction from seeds and is mainly composed of proteins, lignocellulosic fibres and minerals [1]. Rapeseed has become the world’s third largest source of vegetable oil after soy and palm. China produced about 8.2 million tons of rapeseed cake/meal in 2013, only a small part of which qualifies as double-low rapeseed meal (i.e. low erucic acid and low glucosinolate (Gls)). Rapeseed cake/meal, a good protein resource for animal feed, has an excellent balance of essential amino acids and is relatively high in sulfur-containing amino acids. However, the digestibility of RSC for non-ruminant animals is poor due to the presence of anti-nutritional substances such as Gls, phytic acid, tannins etc. [2]. The main toxic compound in the rapeseed meal is Gls. Glucosinolates and its degradation products impair palatability, affect liver and kidney functions, and interfere with iodine availability [3]. The feed ratio of rapeseed meal as protein source remains at lower percentages than that of soybean meal since, most likely, its high content of other anti-nutritional components, such as phenols, phytate and fiber, as well as its lower lysine bioavailability [4]. Moreover, rapeseed proteins are not easily digested compared to other protein rich materials such as fishmeal or soybean meal rendering them less valuable [5].

Various processing techniques, including inactivation of myrosinase, steam stripping, solvent extraction and leaching, etc., have been applied to improve the quality of rapeseed meal, but these methods have several drawbacks such as loss of proteins, cost and commercial unfeasibility, and environmental pollution [6]. Microbial upgrading the quality of rapeseed meal/cake has been researched for a long time. Solid state fermentation (SSF) is the preferred method for the enrichment of agricultural residues since it offers several economical and practical advantages. The SSF systems have been reported to be an effective way to reduce undesired substances of rapeseed meal including phytic acid, Gls and fiber [6-8].

In the present study, SSF was employed to investigate the effect of *A. niger* fermentation on reduction of Gls content of RSC. The chemical composition and amino acid *in vitro* digestibility of FRSC were also assessed under optimal fermentation conditions.

## Materials and methods

### Microorganism and basal substrate

*A. niger* is often used in feedstuff fermentation and has no harmful effects on animals in China. *A. niger* (CICC 41258) was purchased from China Center of Industrial Culture Collection (CICC) and maintained on potato dextrose agar (PDA) slants at 4°C and transferred once every two months. *A. niger* spores were washed from 7-days agar slants with 0.1% Tween 80. The concentration of spores grown in PDA flasks was counted by using a counting chamber.

Rapeseed cake used in the study was obtained from local market in Ya’an, China. The wheat bran was purchased from NanFang Flour Co. Ltd., and RSC and wheat bran were dried in an oven at 105°C to constant weight. Then, they were ground to pass a 40-mesh sieve.

### Solid state fermentation

The experiments were conducted in 250 mL Erlenmeyer flask covered with cotton plugs to facilitate air transfer. The substrates containing RSC and wheat bran, which is beneficial for microbial growth, were sterilized subsequently at 121°C for 20 min, cooled and inoculated with 3 mL spore suspension (1 × 10^7^ spores/mL) of *A. niger*, an uninoculated flask served as control. The samples in flasks were incubated at 30°C for 72 h. After fermentation was completed, inoculated samples were dried at 65°C for 48 h, cooled and ground to pass a 40-mesh sieve for related index determination. Three replicate were prepared for each treatment. All treatments were carried out in two sets.

### Optimization of fermentation process during SSF

Factors including substrate composition, fermentation temperature, incubation time and initial moisture content (IMC), affecting the Gls levels of RSC under SSF, were optimized by adopting a search technique varying parameters one at a time. The experiments were conducted in 250 mL Erlenmeyer flasks containing 30 g basal substrate, 3 mL spore suspension (1 × 10^7^ spores/mL) of *A. niger* was inoculated, pH value was at the nature condition, various experimental conditions are described as follows:

The ratio of RSC to wheat bran: five ratios of RSC to wheat bran (9:1, 8:2, 7:3, 6:4, 5:5) were investigated to study their effects on the Gls contents in RSC. Other conditions were moisture content of solid substrate 50% and incubation at 30°C for 72 h.

Fermented period (hours): various incubation periods (24, 36, 48, 60, 72, 84 and 96 h) were used to evaluate the effects on degradation of Gls in RSC. The fermented solid medium contained RSC 70% and wheat bran 30%, the fermentation was carried out at 30°C.

Incubation temperature: the fermentation was carried out at various temperatures (25, 28, 31, 34 and 37°C) for 72 h to study their influence on degradation of Gls in RSC. All other conditions were kept at optimum levels.

Initial moisture content (IMC): the fermentation was carried out under various IMC (43.0%, 50.0%, 56.0%, 62.0% and 67.5%) with all other parameters kept at their optimum levels.

### Main nutritional components assay

Total Gls was determined according to the palladium chloride [9]. Unfermented RSC and FRSC were analyzed for dry matter, crude protein (CP), ether extract and neutral detergent fiber (NDF) by AOAC International (1990). The trichloroacetic acid soluble protein (TCA-SP) of sample was determined by Ovissipour et al. [10]. The phytic acid content was measured according to the procedure of Nair and Duvnjak [11]. The amino acid (AA) profile of FRSC and unfermented RSC were analyzed using an automatic AA analyzer (L-8900; Hitachi, Tokyo, Japan). Before analysis, samples were hydrolyzed with 6 mol/L HCl at 110°C for 24 h. Methionine and cysteine were analyzed as Met sulfone and cysteic acid after cold performic acid oxidation overnight before hydrolysis.

### *In vitro* digestibility determination

*In vitro* two-stage enzyme hydrolysis procedures were carried out as described by Sakamoto et al. [12], with some modifications. In brief, fermented (or not) RSC substrate (5 g) were placed in 250 mL Erlenmeyer flasks. Thirty mL of a 10,000 U/mL pepsin (activity: 3,000 U/mg, Sigma) solution (0.05 mol/L KCl-HCl buffer, pH 2.0) was blended evenly, incubated at 39°C at 80 revolutions per min for 4 h. Then pH was adjusted to 7.0 with 0.2 mol/L NaOH and 30 mL of 625 U/mL trypsin (activity: 250 U/mg, Sigma) added, blended again, and finally incubated at 39°C at 80 revolutions per min for 4 h. After digestion was complete, the digesta suspension was centrifuged at 2,047 × g for 15 min, the residues were then frozen, freeze-dried and analyzed for subsequent amino acid assay.

### Enzyme activities in the fermenting meal during solid state fermentation

The crude enzymes from the cultured media were extracted with 0.05 mol/L citrate buffer (pH 5.0) and filtered using filter paper. The extract was centrifuged at 10,000 × g for 10 min at 4°C and the supernatant were used as crude enzyme source. Endoglucanase activity was determined using 1% carboxymethyl cellulose in a pH 5 sodium citrate buffer [13]. Xylanase activity was measured by the method of Bailey et al. [14]. One unit of enzyme was defined as the amount of enzyme required to release 1 μmol of glucose for endoglucanase and xylose for xylanase from the appropriate substrates per minute under assay conditions. Phytase activity was measured using sodium phytate as the substrate as described by Harland and Harland [15]. Phytase activity was determined was defined as the amount of enzyme required to release 1 mg inorganic phosphorus per hour at the given temperature. Acid protease activity was determined using 2% hemoglobin dissolved in glycine-HCl (pH 3.2, 0.1 mol/L) as substrate, as reported earlier [16]. A unit of acid protease activity was defined as the amount of enzyme liberating 1 μg tyrosine per minute at the incubation temperature of 40°C.

### Statistical analysis

One-way analysis of variance was performed using the General Linear Models Procedure of the SAS software (SAS, 1999). Differences among means were tested using Duncan’s multiple range tests. Differences between two means were tested using Student’s T-test. A significant level of 0.05 was used as indication of a difference.

## Results

### Optimization of fermentation process on biodegradation of Gls

**Figure 1:**
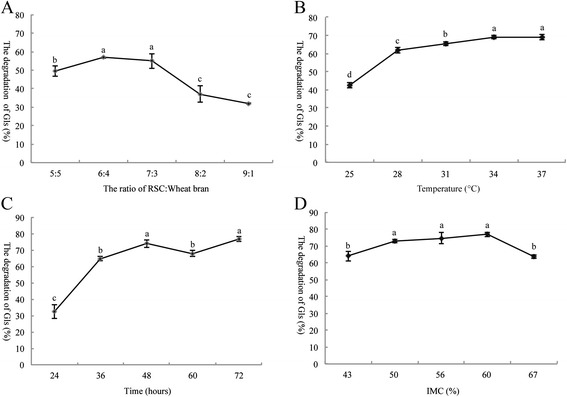
**Effect of fermentation conditions and composition of meal on the reduction of Gls levels. (A)** the ratio of RSC to wheat bran; **(B)** the temperature; **(C)** the incubation time; **(D)** the initial moisture content (IMC).

Five ratios of RSC to wheat bran (5:5, 6:4, 7:3, 8:2 and 9:1) were used to study their effects on the degradation rate of Gls in RSC (Figure 1A). When the ratio was 6:4 or 7:3, *A. niger* cell grew well and degradation rate of Gls significantly higher (*P* < 0.05) than 5:5. When RSC content increased to 80% or 90%, both filamentous fungi growth and the degradation rate of Gls (36.98% and 31.97%) markedly decreased (*P* < 0.05). The above results showed the optimum ratio of RSC to wheat bran in solid medium was 6:4 or 7:3. Fermented medium containing 70% RSC was selected and used for subsequent studies.

The optimal temperature for the degradation rate of Gls was found to be 34°C (Figure 1B), indicating this temperature being favorable for *A. niger* growth in RSC medium. The degradation rate in FRSC was 69.16% at 34°C, markedly higher (*P* < 0.05) than at 28°C and 31°C, but there was no significant difference (*P* > 0.05) with FRSC at 37°C.

Studies found that the Gls was different during the changes of fermentation periods. After 24 h incubation, the degradation rate of Gls was 32.45%, with the fermentation prolonged, the degradation rate of Gls increased. The maximum degradation rate (76.84%) was attained at 72 h (Figure 1C), but there were no significant difference (*P* > 0.05) in degradation rate between 48 and 72 h incubation. 72 h incubation time was selected for subsequent studies.

The water content of the substrate plays an important role in both microorganism growth and enzyme production in SSF. Figure 1D showed the substrate containing 60% moisture achieved the degradation rate of Gls at levels of 76.89%. The optimal IMC for degradation rate of Gls appeared to be 50–60%. Initial moisture content (IMC) above 60% or below 50% both markedly decreased (*P* < 0.05) the degradation rate of Gls.

### Changes in chemical composition during solid state fermentation

In the present study, after 72 h of incubation at 34°C, the TCA-SP of FRSC was 15.93% at IMC 60% (Table 1). Compared to unfermented RSC meal, the TCA-SP of FRSC increased (*P* < 0.05) by 104%. The CP content of FRSC was significantly higher (*P* <0.05) than the unfermented RSC except for the 1-d time point. The ether extract contents in 1–3 d FRSC were significantly higher (*P* < 0.05) than those in unfermented RSC. Compared to the unfermented group, the NDF contents was reduced (*P* < 0.05) by 9.12% after 3-d fermentation with *A. niger*, but there were no significant difference (*P* > 0.05) between 1-d FRSC and unfermented group. After 3 d fermentation with *A. niger*, phytic acid content in FRSC was reduced (*P* < 0.05) by 44.60%.

**Table 1:** **Effect of** ***A. niger*** **fermentation on components of rapeseed cake, as-DM basis** ^**1**^

**Contents, %**	**Control**	**FRSC** ^**3**^			**SEM** ^**4**^
		**Incubation time**			
	**(RSC)** ^**2**^				
		**1d**	**2d**	**3d**	
Crude protein	31.84^c^	33.70^bc^	38.05^ab^	39.17^a^	1.11
TCA-SP^5^	7.82^d^	9.69^c^	12.48^b^	15.93^a^	0.94
Ether extract	5.14^c^	5.72^b^	6.07^ab^	6.35^a^	0.14
NDF	38.39^a^	38.52^a^	34.19^b^	34.89^b^	0.68
Phytic acid	2.78^a^	2.78^a^	2.55^b^	1.54^c^	0.15

^1^Values are means of three replicates per treatment. Means in a row with no common letters differ significantly (*P* <0.05).

^2^RSC = rapeseed cake.

^3^FRSC = fermented rapeseed cake.

^4^SEM = Standard error of the mean.

^5^TCA-SP = trichloroacetic acid soluble protein.

### Amino acid assay of FRSC

Changes in AA content induced by fermentation are presented in Table 2. The CP content of the FRSC was markedly improved by 73.3 g/kg DM, compared to the unfermented group, and the total amino acids (TAA) and essential amino acids (EAA) of fermented substrate also increased by 63.2 and 26.1 g/kg DM, respectively (Table 2). Compared to the control, the levels of methionine and threonine were improved (*P* < 0.05) by 2.26 and 4.95 g/kg DM, respectively, after fermentation, but there were no significant difference (*P* > 0.05) in lysine between unfermented RSC and FRSC. However, the histamine content of the FRSC was reduced (*P* < 0.05) by 3.32 g/kg DM.

**Table 2:** **Crude protein (CP) and amino acid (AA) content of RSC or FRSC** ^**1**^

**Nutrients**	**Control (RSC)** ^**2**^	**FRSC** ^**3**^	**SEM** ^**4**^
CP	318.40^b^	391.70^a^	11.10
Ala	15.29^b^	20.02^a^	1.13
Arg*	10.96^b^	14.95^a^	0.97
Asp	20.18^b^	28.59^a^	1.94
Cys	5.96^a^	7.63^b^	0.86
Glu	59.73	66.32	1.98
Gly	15.96^b^	19.85^a^	1.02
His*	18.53^a^	15.21^b^	0.99
Ile*	10.67^b^	14.84^a^	0.98
Leu*	18.74^b^	24.70^a^	1.37
Lys*	17.29	17.84	1.28
Met*	4.60^b^	6.86^a^	0.58
Phe*	12.52^b^	18.02^a^	1.27
Pro	18.33^b^	23.06^a^	1.28
Ser	13.90^b^	17.91^a^	0.93
Thr*	13.32^b^	18.27^a^	1.14
Tyr	5.97^b^	9.60^a^	0.92
Val*	16.02^b^	21.87^a^	1.38
Total AA	277.95^b^	345.54^a^	14.39
Essential AA	122.64^b^	157.56^a^	6.84

^1^Values are means of three replicates per treatment. Means in a row without common superscript differ significantly (*P* <0.05). Crude protein and amino acid contents are expressed as g/kg DM.

^2^RSC = rapeseed cake.

^3^FRSC = fermented rapeseed cake.

^4^SEM = Standard error of the mean.

^*^The amino acid is an essential amino acid.

### *In vitro* amino acid digestibility of FRSC

The results of *in vitro* AA digestibility of RSC fermented by *A. niger* are presented in Table 3. *In vitro* TAA and EAA digestibility of FRSC was improved (*P* < 0.05) by 5.87 and 6.69 percentage units respectively, compared with unfermented substrate (control). In addition, the *in vitro* digestibility of nine amino acids including four essential amino acid (methionine, lysine, arginine and histamine) was also improved greatly (*P* < 0.05).

**Table 3:** ***In vitro*** **AA digestibility (%) of RSC fermented by** ***A. niger*** **, as-DM basis** ^**1**^

**Items**	**RSC (Control)** ^**2**^	**FRSC** ^**3**^	**SEM** ^**4**^
Ala	62.46^b^	68.15^a^	1.44
Arg*	73.96^b^	79.52^a^	1.36
Asp	69.03	70.39	0.54
Cys	71.68^b^	76.83^a^	1.30
Glu	75.60	78.28	0.76
Gly	62.40^b^	65.79^a^	0.91
His*	66.47^b^	70.19^a^	1.99
Ile*	66.19	63.60	1.03
Leu*	61.22	66.38	1.56
Lys*	71.20^b^	78.61^a^	1.70
Met*	76.42^b^	82.24^a^	1.43
Phe*	66.14	69.16	2.80
Pro	56.58^b^	64.01^a^	1.76
Ser	59.45^b^	65.87^a^	1.62
Thr*	59.89	63.95	1.22
Tyr	58.97^b^	63.75^a^	1.28
Val*	65.06	67.26	1.00
Total AA	67.14^b^	71.08^a^	1.09
Essential AA	64.90^b^	69.24^a^	1.20

^1^Values are means of three replicates per treatment. Means in a row without common superscript differ significantly (*P* <0.05).

^2^RSC = rapeseed cake.

^3^FRSC = fermented rapeseed cake.

^4^SEM = Standard error of the mean.

^*^The amino acid is an essential amino acid.

### Enzyme production during solid state fermentation

**Figure 2:**
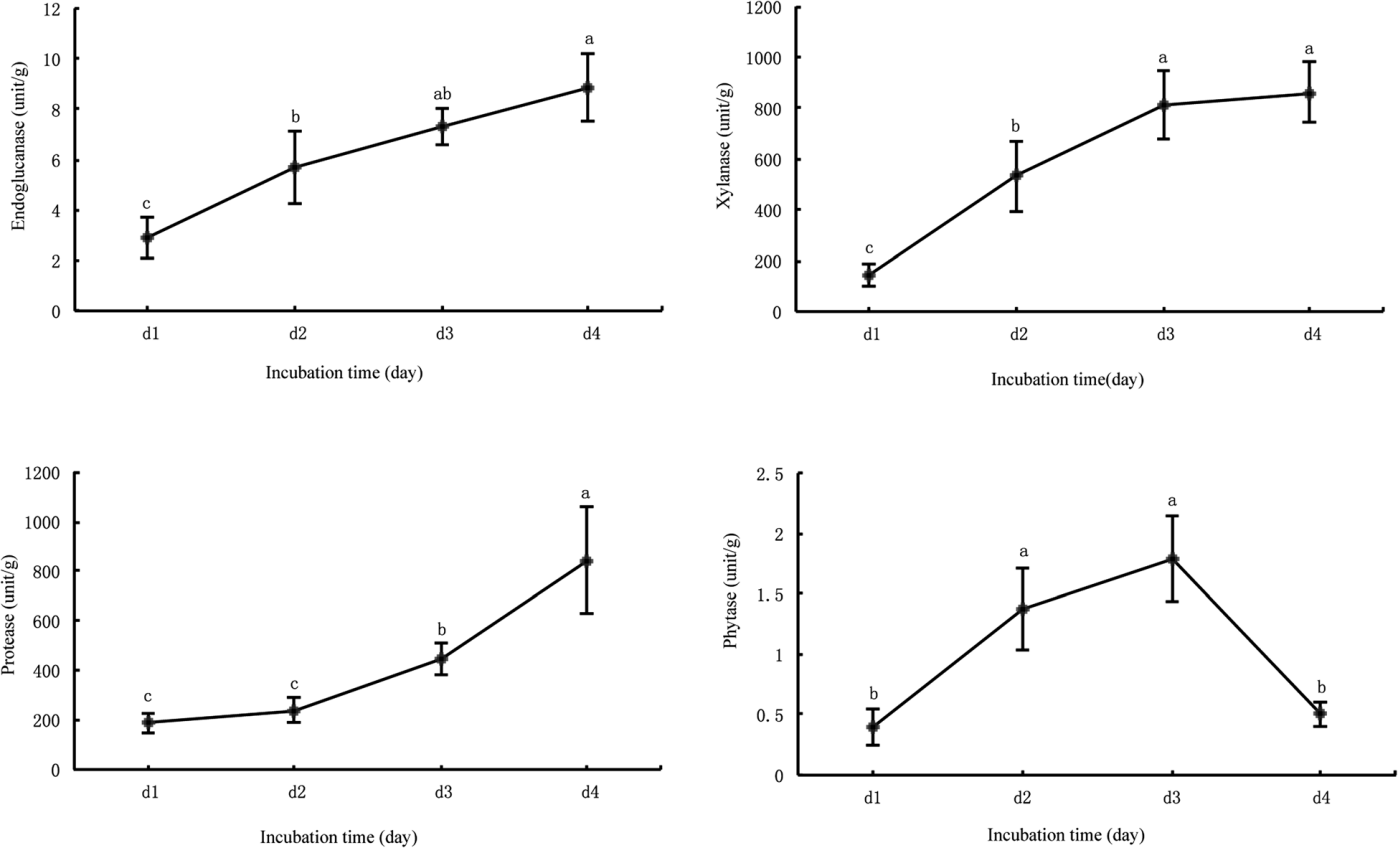
**Endoglucanase, xylanase, acid protease and phytase activities in the fermenting meal during SSF.**

Multiple enzymes are required to eliminate the anti-nutritional components and improve the protein quality in the RSC. Several enzyme activities (endoglucanase, xylanase, acid protease and phytase) of 1–4 day fermented RSC by *A. niger* were determined (Figure 2). With the fermentation prolonged, endoglucanase, xylanase, acid protease activity also increased (*P* < 0.05) from 24 to 96 h. After 3 day’s fermentation, maximal phytase protuction (1.79 unit/g) in FRSC was obtained, and then phytase activity of 4 d-FRSC markedly decreased (*P* < 0.05).

## Discussion

Rapeseed meal has a high protein content (about 35%) with an excellent balance of essential amino acids, but its use in animal feed is generally limited by the presence of toxic or indigestible components including Gls, phytic acid, some ethanol-soluble carbohydrates and polyphenols [8]. Major deleterious effects of Gls ingestion in animals are reduced palatability, decreased growth and production. Therefore, a desired amount of RSC can be used for animal feed formulation adopting a suitable technology to minimize Gls-related deleterious effects on animal [3]. The use of solid-state fermentation for upgrading the nutritional characteristics of raw plant materials has been proposed to improve their use in animal feeds. The present results showed that Gls content in FRSC was markedly influenced by the medium composition and incubation conditions. When the fermentation substrate was 100% RSC, *A. niger* grew very slowly (data not shown). The ratio of carbon to nitrogen (C/N) in RSC might not be a suitable level for the growth of *A. niger*. Wheat bran, as a carbon sources, is beneficial to loosen solid medium and overcoming the agglomeration of the substrate [17]; therefore, suitable ratio of wheat bran was necessary to support better microbial fermentation. In the present study, optimum ratio of RSC to wheat bran was 7:3 or 6:4 for the degradation of Gls of FRSC. In addition to composition of fermentation substrate, several fermentation conditions included IMC, temperature and incubation time, affected biodegradation rate of Gls. In the previous study, a 40-h fermentation with *Rhizopus oligosporus* resulted in the degradation of 47% of total Gls in RSM [8]. Rakariyatham and Sakorn [18] showed that 100 μmol/g of Gls was totally degraded by SSF with *Aspergillus sp.* NR-4201 in 48 h. In the present study, maximal biodegradation rate of Gls was 76.89% at optimal fermented conditions. The degradation of Gls may be due to utilization of glucose and sulphur moieties of these compounds by microbial enzymes [3].

*A. niger* fermented RSC also exhibited an increase in TCA-SP, CP and ether extract with an increase in incubation time. Trichloroacetic acid soluble nitrogen, which is assumed to consist only of small peptides (2–20 residues) and free amino acids [19], is easily absorbed in the animal's gut system. In the present study, the TCA-SP of FRSC also significantly increased with the fermentation prolonged. Solid stated fermentation with *Aspergillus oryzae* had been conducted to convert rapeseed meal proteins into free amino nitrogen [4,5]. An increased amount of TCA-SP in FRSC is due to partial digestion of large-size peptides in RSC by proteases secreted by *A. niger* during fermentation. The loss of dry matter at the expense of fermentable sugars during fermentation could be a possible reason for an increase in nitrogen [8]. Hong et al. (2004) demonstrated that crude fat contents in soybeans and soybean meal also increased after A. *oryzae* fermentation [20]. The increases in fat contents may be partly caused by the decreased carbohydrate content after fermentation. Carbohydrates in RSC may be used as substrates for energy and synthesis of fatty acids.

After fermentation for 72 h, except for Gls, other anti-nutritional compounds including NDF and phytic acid in FRSC also markedly declined. This result also was confirmed by several authors. Vig and Walia (2001) investigated the effect of R. *oligosporus* fermentation on the components of rapeseed meal. The results showed that glucosinolates, phytic acid and crude fiber were reduced by 43.1%, 42.4% and 25.5%, respectively, after fermentation [6]. Wang et al. (2012) used composite strains to eliminate the anti-nutritional substances in rapeseed meal, the phytic acid and crude fiber were reduced by 28.3% and 66.2%, respectively [21]. In the present study, the reduction of glucosinolates, phytate and NDF by fermentation might due to the production of relevant enzyme by *A. niger* which cause the breakdown of anti-nutritional substrates.

Since crude protein increased after fermentation, the amino acid content of fermented RSC would also be increased except His. Khalaf and Meleigy (2008) reported that total amino acids (TAA) and essential amino acids (EAA) of fermented cottonseed meal increased by 22.4 and 9.6 g/kg DM, respectively [22]. In the present study, they were increased by 62.58 and 29.92 g/kg DM, respectively. These changes may be attributed to higher increase in protein content (73.3 g/kg DM) in our study than that by Khalaf and Meleigy (2008). The histamine content of FRSC was reduced by 3.32 g/kg DM, which may be attributable to the preferential utilization of histamine by *A. niger*.

An increase in AA digestibility after fermentation in RSC substrate was observed in the present study. The *in vitro* digestible CP and AA of cotton seed meal (CSM) fermented by *C. tropicalis* ZD-3 was increased significantly compared with unfermented CSM [23]. However, CP and AA digestibility in FRSC is scarce to date. The AA *in vitro* digestibility increase was mainly associated with growth of *A. niger,* which has ability to secrete many extra-cellular degradation enzymes, especially protease.

Since 1990, several papers have described the use of enzymatic hydrolysis treatments (proteases, glycosyl hydrolases) on RSM to increase nutritional value and digestibility [24-26]. The use of RSM as main substrate for phytase, myrosinase producing by *Aspergillus* sp. had been reported [27,28]. In the present study, several enzyme activities (endoglucanase, xylanase, acid protease and phytase) of 1–4 day fermented RSC by *A. niger* were determined. With the fermentation prolonged, the activity of these enzymes also significantly increased. An increase in TCA-SP and AA *in vitro* digestibility of FRSC might be caused by hydrolysis of proteins with acid protease from *A. niger*. The decrease of Gls, NDF, phytic acid might be resulted from hydrolysis by relevant enzyme.

## Conclusions

Solid state fermentation with *A. niger* can effectively reduce Gls levels in RSC and increase the TCA-SP, CP, ether extract contents. Furthermore, the *in vitro* AA digestibility was improved via SSF in this study. In our recent study found that the performance and total tract nutrient digestibility was improved for growing pigs fed diet including 10% FRSC compared with unfermented RSC and was similar to the performance obtained with pigs fed corn-soybean meal diet (unpublished data). Therefore, FRSC can be used as a new feed ingredient in animal diet, especially for non-ruminant animals. Our results suggest that the SSF method offers an effective approach to improving the quality of unconventional proteins sources such as the RSC.

## Abbreviations

SSF: Solid stated fermentation
RSC: Rapeseed cake
Gls: Glucosinolates
FRSC: Fermented rapeseed cake
CP: Crude protein
TCA-SP: Trichloroacetic acid soluble protein
DM: Dry matter
AA: Amino acid
NDF: Neutral detergent fiber

## References

[1] Lomascolo A, Uzan-Boukhris E, Sigoillot JC, Fine F (2012). Rapeseed and sunflower meal: a review on biotechnology status and challenges. Appl Microbiol Biotechnol.

[2] Khajali F, Slominski BA (2012). Factors that affect the nutritive value of canola meal for poultry. Poult Sci.

[3] Tripathi MK, Mishra AS (2007). Glucosinolates in animal nutrition: A review. Anim Feed Sci Tech.

[4] Wang R, Shaarani SM, Godoy LC, Melikoglu M, Vergara CS, Koutinas A (2010). Bioconversion of rapeseed meal for the production of a generic microbial feedstock. Enzyme Microb Technol.

[5] Uckun KE, Salakkam A, Trzcinski AP, Bakir U, Webb C (2012). Enhancing the value of nitrogen from rapeseed meal for microbial oil production. Enzyme Microb Technol.

[6] Vig AP, Walia A (2001). Beneficial effects of Rhizopus oligosporus fermentation on reduction of glucosinolates, fibre and phytic acid in rapeseed (Brassica napus) meal. Bioresource technol.

[7] Bau H, Villaume C, Lin C, Evrard J, Quemener B, Nicolas J (1994). Effect of a solid-state fermentation using Rhizopus oligosporus sp.T-3 on elimination of antinutritional substances and modification of biochemical constituents of defatted rapeseed meal. J Sci Food Aguic.

[8] Rozan P, Villaume C, Bau H, Schwertz A, Nicolas J, Méjean L (1996). Detoxication of rapeseed meal by Rhizopus Oligosporus sp-T3: A first step towards rapeseed protein concentrate. Int J Food Sci Tech.

[9] Wathelet JP, Wagstaffe PJ, Biston R, Marlier M, Severin M (1988). Rapeseed reference materials for glucosinolate analysis. Fresenius J Anal Chem.

[10] Ovissipour M, Abedian A, Motamedzadegan A, Rasco B, Safari R, Shahiri H (2009). The effect of enzymatic hydrolysis time and temperature on the properties of protein hydrolysates from Persian sturgeon (Acipenser persicus) viscera. Food Chem.

[11] Nair VC, Duvnjak Z (1990). Reduction of phytic acid content in canola meal by Aspergillus ficuum in solid state fermentation process. Appl Microbiol Biotechnol.

[12] Sakamoto K, Asano T, Furuya A, Takahashi S (1980). Estimation of in vivo digestibility with the laying hen by an in vitro method using the intestinal fluid of the pig. Brit J Nutr.

[13] Ghose TK (1987). Measurement of cellulase activities. Pure Appl Chem.

[14] Bailey MJ, Biely P, Poutanen K (1992). Interlaboratory testing of methods for assay of xylanase activity. J Biotechnol.

[15] Harland B, Harland J (1980). Fermentative reduction of phytic acid in rye, white and whole wheat bead. Cereal Chem.

[16] Tello-Solís S, Rodríguez-Romero A, Hernández-Arana A (1994). Circular dichroism studies of acid proteinases from Aspergillus niger and Aspergillus awamori. Biochem Mol Biol Int.

[17] Weng X, Sun J (2006). Biodegradation of free gossypol by a new strain of Candida tropicalis under solid state fermentation: Effects of fermentation parameters. Proc Biochem.

[18] Rakariyatham N, Sakorn P (2002). Biodegradation of glucosinolates in brown mustard seed meal (Brassica juncea) by Aspergillus sp. NR-4201 in liquid and solid-state cultures. Biodegradation.

[19] Kuchroo C, Fox P (1982). Soluble nitrogen in Cheddar cheese: Comparison of extraction procedures. Milchwissenschaft.

[20] Hong KJ, Lee CH, Kim SW (2004). Aspergillus oryzae GB-107 fermentation improves nutritional quality of food soybeans and feed soybean meals. J Me Food.

[21] Jin XS, Wang QZ, Wang T, Huang JH, Xia YX, Yao LX (2012). Screening of glucosinolate-degrading strains and its application in improving the quality of rapeseed meal. Ann Microbiol.

[22] Khalaf MA, Meleigy SA (2008). Reduction of free gossypol levels in cottonseed meal by microbial treatment. Int J Agric Biol.

[23] Zhang WJ, Xu ZR, Zhao SH, Sun JY, Yang X (2007). Development of a microbial fermentation process for detoxification of gossypol in cottonseed meal. Anim Feed Sci Tech.

[24] Mahajan A, Dua S (1998). Improvement of functional properties of rapeseed (Brassica campestris var toria) meal by reducing antinutritional factors employing enzymatic modification. Food Hydrocolloid.

[25] Fang ZF, Peng J, Liu ZL, Liu YG (2007). Responses of non-starch polysaccharide-degrading enzymes on digestibility and performance of growing pigs fed a diet based on corn, soya bean meal and Chinese double-low rapeseed meal. J Anim Physiol An N.

[26] Fang ZF, Peng J, Tang TJ, Liu ZL, Dai JJ, Jin LZ (2007). Xylanase supplementation Improved Di gestibility and performance of growing pigs fed Chinese double-low rapeseed meal inclusion diets: in vitro and in vivo studies. Asian-Aust J Anim Sci.

[27] Ebune A, Al-Asheh S, Duvnjak Z (1995). Production of phytase during solid-state fermentation using Aspergillus Ficuum NRRL 3135 in canola meal. Bioresource Technol.

[28] Butrindr B, Niamsup H, Shank L, Rakariyatham N (2004). Myrosinase overproducing mutants of Aspergillus sp. NR463. Ann Microbiol.

